# Trial of Repeated Analgesia with Kangaroo Mother Care (TRAKC Trial)

**DOI:** 10.1186/1471-2431-13-182

**Published:** 2013-11-09

**Authors:** Marsha Campbell-Yeo, Celeste Johnston, Britney Benoit, Margot Latimer, Michael Vincer, Claire-Dominique Walker, David Streiner, Darlene Inglis, Kim Caddell

**Affiliations:** 1 School of Nursing, Dalhousie University, Halifax, NS, Canada; 2 Department of Pediatrics, IWK Health Centre, Halifax, NS, Canada; 3 Centre for Pediatric Pain Research, IWK Health Centre, Halifax, NS, Canada; 4 School of Nursing, McGill University, Montreal, QC, Canada; 5 Neuroscience Research Division, Douglas Institute, Montreal, QC, Canada; 6 Department of Psychiatry, University of Toronto, Toronto, ON, Canada; 7 Department of Psychiatry and Behavioural Neurosciences, McMaster University, Toronto, ON, Canada; 8 Maternal Newborn Program, IWK Health Centre, Halifax, NS, Canada

**Keywords:** Neonatal pain, Skin-to-skin contact, Kangaroo Mother Care, Neonatal Intensive Care Unit, Preterm neonates, Sucrose, Randomized controlled trial

## Abstract

**Background:**

Skin-to-skin contact (SSC) between mother and infant, commonly referred to as Kangaroo Mother Care (KMC), is recommended as an intervention for procedural pain. Evidence demonstrates its consistent efficacy in reducing pain for a single painful procedure. The purpose of this study is to examine the sustained efficacy of KMC, provided during all routine painful procedures for the duration of Neonatal Intensive Care Unit (NICU) hospitalization, in diminishing behavioral pain response in preterm neonates. The efficacy of KMC alone will be compared to standard care of 24% oral sucrose, as well as the combination of KMC and 24% oral sucrose.

**Methods/design:**

Infants admitted to the NICU who are less than 36 6/7 weeks gestational age (according to early ultrasound), that are stable enough to be held in KMC, will be considered eligible (N = 258). Using a single-blinded randomized parallel group design, participants will be assigned to one of three possible interventions: 1) KMC, 2) combined KMC and sucrose, and 3) sucrose alone, when they undergo any routine painful procedure (heel lance, venipuncture, intravenous, oro/nasogastric insertion). The primary outcome is infant’s pain intensity, which will be assessed using the Premature Infant Pain Profile (PIPP). The secondary outcome will be maturity of neurobehavioral functioning, as measured by the Neurobehavioral Assessment of the Preterm Infant (NAPI). Gestational age, cumulative exposure to KMC provided during non-pain contexts, and maternal cortisol levels will be considered in the analysis. Clinical feasibility will be accounted for from nurse and maternal questionnaires.

**Discussion:**

This will be the first study to examine the repeated use of KMC for managing procedural pain in preterm neonates. It is also the first to compare KMC to sucrose, or the interventions in combination, across time. Based on the theoretical framework of the brain opioid theory of attachment, it is expected that KMC will be a preferred standard of care. However, current pain management guidelines are based on minimal data on repeated use of either intervention. Therefore, regardless of the outcomes of this study, results will have important implications for guidelines and practices related to management of procedural pain in preterm infants.

**Trial registration:**

ClinicalTrials.gov Identifier: NCT01561547.

## Background

Even in the context of critical care, mothers can provide comfort to their preterm neonates during painful procedures. Skin-to-skin contact (SSC), commonly referred to as Kangaroo Mother Care (KMC), is recommended as an intervention for procedural pain in guidelines by the Canadian Pediatric Society and the American Academy of Pediatrics [1]. There are now more than 18 studies showing consistent efficacy of KMC in reducing pain from a single painful procedure [2,3]. These same guidelines also recommend 24% oral sucrose for common procedural pain based on more than 57 studies [4]. Despite some concerns with the influence of sucrose on neurodevelopmental outcomes [5,6], sucrose is considered standard care for management of procedural pain in most Neonatal Intensive Care Units (NICUs) [7].

It is assumed that KMC, shown to be effective for managing pain in neonates during single events, will remain efficacious over time. However, as no studies have examined its repeated use, this is unknown. In addition, as the mechanism underlying oral sucrose and KMC are thought to differ (sweet taste is believed to release endorphins and KMC believed to release oxytocin), the interventions could have synergistic effects when used in combination. The purpose of this study is to examine the sustained efficacy of KMC in diminishing pain response in preterm neonates compared to standard care of oral sucrose, as well as examine the combination of KMC and oral sucrose. Infant’s neurodevelopment at term equivalence will also be compared between groups.

## Summary of literature review

### Pain in the NICU

More than two decades ago, two Canadian surveys examining pain and its management in the NICU reported under-treatment of procedural pain [8,9]. These surveys indicated that unlike post-operative pain, which is typically treated with analgesic intervention, management of procedural pain using analgesia was uncommon. In these surveys, the number of tissue damaging procedures was documented as averaging from 2 to 8 per infant [8]. The findings of these Canadian surveys are consistent with the findings of surveys conducted in the US [10,11] and UK [12].

More recently, surveys conducted in Canadian, Dutch, and French NICUs indicate that infants undergo a similar number of procedures with almost 50% of all tissue breaking procedures associated with little or no analgesia [13-15]. The under management of pain noted within the literature has been reported in spite of guidelines recommending analgesia and comfort measures in neonates undergoing medical procedures [1], as well as the fact that most neonatal staff believe that infants are capable of experiencing pain from a very early age [13].

### Treatment of procedural pain

#### *Pharmacologic agents*

Pharmacologic agents that have been examined for reducing procedural pain in neonates include topical anesthetics and systemic drugs, such as opioids or acetaminophen. Topical anesthetics, while effective for pain management during circumcision [16-20], have been shown to be ineffective for heel lance, venipuncture, and insertion of intravenous lines [21-25]. Systemic drugs, specifically opiates, are highly sensitive to the developmental stage of the infant [26,27]. Therefore, rapid changes in requirements make effective and safe dosing a challenge. In addition, opiates have significantly slower clearance in neonates [28-32], and have not necessarily been demonstrated as effective for managing procedural pain [33]. Trials of acetaminophen for procedural pain control have demonstrated that it is not effective, and may even be no better than placebo for heel lance procedure [34,35]. Thus, pharmacological management for common, repeated, painful procedures in preterm neonates is not an option. Given the frequency of painful procedures in NICUs, the short and long term negative effects of repeated pain exposure in this population, and the ethical imperative to manage this pain, other approaches are required.

#### *Alternative methods of procedural pain control*

Non-pharmacological approaches to pain management are thought to be based on the release of endogenous opiates [36,37]. Although there are scant data in neonates regarding endogenous descending inhibitory mechanisms, the engagement of mechanisms that release endorphins is well established in adults [36,37]. Animal studies suggest that the endogenous system is not well developed prior to 32-weeks post-conception, however, there is speculation that it may be developed enough to provide some level of comfort [38,39]. Various non-pharmacological strategies for pain control have been tested in neonates and show varying degree of efficacy. These strategies can be categorized into sensory stimulation [40,41] (i.e., positioning/swaddling, vestibular action/rocking, non-nutritive sucking, music), nutritive [42-63] (i.e., breastfeeding, oral sweet solutions), and maternal interventions (i.e., maternal odor and voice, breastfeeding, KMC). For the purposes of this trial, KMC and 24% oral sucrose (considered standard care) are the interventions of interest.

#### *Sucrose*

Research in animals and human infants have shown that intra-oral sucrose solutions have an analgesic effect. Studies using sucrose for pain relief started in the late 1980’s [50-52] and have since included both term and preterm infants. Sucrose, the oral solution used most frequently, has been examined in two systematic reviews [50,62], and one meta-analysis [4]. These reviews are in agreement regarding the positive efficacy of oral sucrose for reducing procedural pain in preterm neonates.

Infant response to interventions has been assessed using both behavioral (e.g., cry and facial action) and physiological indicators, as well as composite measures of pain. A recent study demonstrated that although sucrose decreased scores on the Premature Infant Pain Profile (PIPP), there was still nociceptive activity in the somatosensory cortex [63]. Given the dissociation between the affective components of pain [64-66], that is how much it hurts (somatosensory cortex) vs. how aversive it is (frontal and pre-frontal cortex), it is likely that it is the affective component of the pain response that is being modified by sucrose. The authors of the study thus raised concerns about the analgesic properties of sucrose, and whether it can ameliorate the adverse effects of repeated pain exposure. Such findings reinforce the need for continued study of other non-pharmacological interventions such as KMC alone or in conjunction with sucrose.

The effect of sucrose on pain intensity on consecutive days has been addressed, but needs further investigation. While one study examining the use of sucrose for all painful procedures in the first week of life in the NICU reported poorer neurodevelopmental outcome scores with higher doses of sucrose [6], a secondary analysis found that this was only the case in infants who received more than 10 doses over 24 hours [67]. Concerns about the long-term use of sucrose on neurodevelopmental outcomes have also been raised by Holsti and Grunau [5]. They provide a strong and logical argument that the development of the infant’s dopaminergic system is altered with sucrose administration. Specifically, that sugar stimulates dopamine release, and that this release responds or is a reaction to the concentration of sucrose rather than the volume. In addition to this argument, there is also a relationship between dopamine and attention and motor development – the specific neurodevelopmental outcomes used in the study examining repeated sucrose administration [6]. Holsti and Grunau suggest that dopamine regulation is disturbed by receiving repeated doses of 24% sucrose (the recommended concentration for procedural pain management in neonates), and that this interferes with attention and motor development.

#### *Kangaroo Mother Care*

Kangaroo Mother Care (KMC) was implemented in the modern era as an alternative to incubator care to maintain preterm infants’ body temperature and increase survival rates in South America due to short supply of incubators [68]. During this time, it was serendipitously noted that infants in KMC spent more time in quiet sleep state [69,70]. As quiet sleep state is associated with decreased pain response [71,72], the idea developed to use KMC to control procedural pain. When initially studied in full term neonates, decreased crying and heart rate acceleration was observed [73]. Subsequent studies in preterm neonates showed decrease in facial action, decrease in heart rate acceleration, and increased oxygen saturation, with a decrease in overall pain scores [74].

In a recent Cochrane review of the literature on skin-to-skin contact for procedural pain in infants conducted by the authors of this protocol [3], 13 studies that met the inclusion criteria showed positive results. Interestingly, two of the studies [75,76] showed that KMC was more efficacious than sweet taste in reducing infant’s procedural pain. However, to our knowledge, there have been no published studies to date examining the combined efficacy of KMC and sucrose in managing procedural pain in preterm infants.

**Mechanism underlying KMC as a comfort strategy** There are likely several mechanisms underlying the specific pain-relieving effect of KMC. One hypothesis that could be related to all non-pharmacological strategies is derived from the Gate Control Theory of Melzack and Wall [36]. According to this theory, stimuli travelling ascending pathways may inhibit the nociceptive signals from painful stimulus through various endogenous mechanisms located along the spino-thalamic tract [77]. The stronger these competing stimuli are, including multiple modalities, the more effective they are at blocking the perception of pain. Such theories help explain why multiple modalities, such as KMC, breastfeeding, or sensorial saturation that involves tactile, auditory, and olfactory mechanisms are more effective than single modalities.

The mechanism of the comforting effect of breastfeeding and KMC is likely related to the release of oxytocin [78,79]. Oxytocin has been referred to as the love hormone due to its role in affiliative behaviors [80-83]. In an animal model of pain and oxytocin, massage-like stroking induced acute antinociception that was reversed by an oxytocin antagonist [84]. Also, when oxytocin was injected into the periaqeductal gray region, it had an antinociceptive effect.

In summary, 1) procedural pain in the NICU is infrequently and poorly managed, 2) pharmacological interventions present problems with this population, 3) among non-pharmacological interventions both sucrose and KMC are supported by numerous studies as one-time interventions in diminishing pain response, 4) there are no studies of repeated use of KMC and only two studies that have followed infants for repeated use of sucrose over time have had conclusive results, 5) no published studies to date have examined the combination of KMC and sucrose across time.

Given the findings of research to date, this study proposes that KMC alone or in combination with sucrose will continue to reduce pain across the NICU stay more than sucrose alone. This is based on the belief that the touch and olfactory senses invoked from the mother are fundamental to the maternal role of affiliation and comfort through underlying mechanisms of hormone release. It is further proposed that neurodevelopment will be better in infants who have received KMC for minor painful procedures due to the release of norepinephrine during KMC.

### Theoretical framework

Nelson and Panksept’s Brain Opioid Theory of Social Attachment provides the conceptual framework for this study [79]. This theory, based on animal experimentation, postulates that maternal touch, smell, (and milk) release endogenous opiates, which are known to reduce pain and promote affiliative behaviors. There are other hormones involves in mother-infant attachment and comforting, namely oxytocin and epinephrine. Oxytocin, as described above, is the primary hormone promoting affiliation, is released with social contact, and also appears to have antinociceptive effects [84].

### Hypotheses

The hypotheses of this study are as follows:

i. Pain response to heel lance or venipuncture for blood sampling, as measured by the Premature Infant Pain Profile (PIPP), will be lower by 1.5 points in KMC alone than sucrose alone. The combination will be marginally better (0.5 on PIPP scale) for a total of 2 points between combination and sucrose alone and 0.5 points between KMC alone and combination.

ii. This difference in PIPP scores will remain across the infant’s hospitalization.

iii. Neurodevelopmental sub-scores of the Neurobehavioral Assessment of the Preterm Infant (NAPI) of 1) motor development and vigor and 2) alertness and orientation at discharge from the NICU will be significantly higher in infants in the KMC groups than the sucrose alone group.

A no-treatment control group will not be used in this study, as it is believed to be unethical given that it has been argued that a state of equipoise has been reached with sucrose [85], and it is considered the standard of care. According to the hypotheses listed above, it is proposed that KMC will be a preferred standard of care.

## Methods

### Trial design

**Figure 1:**
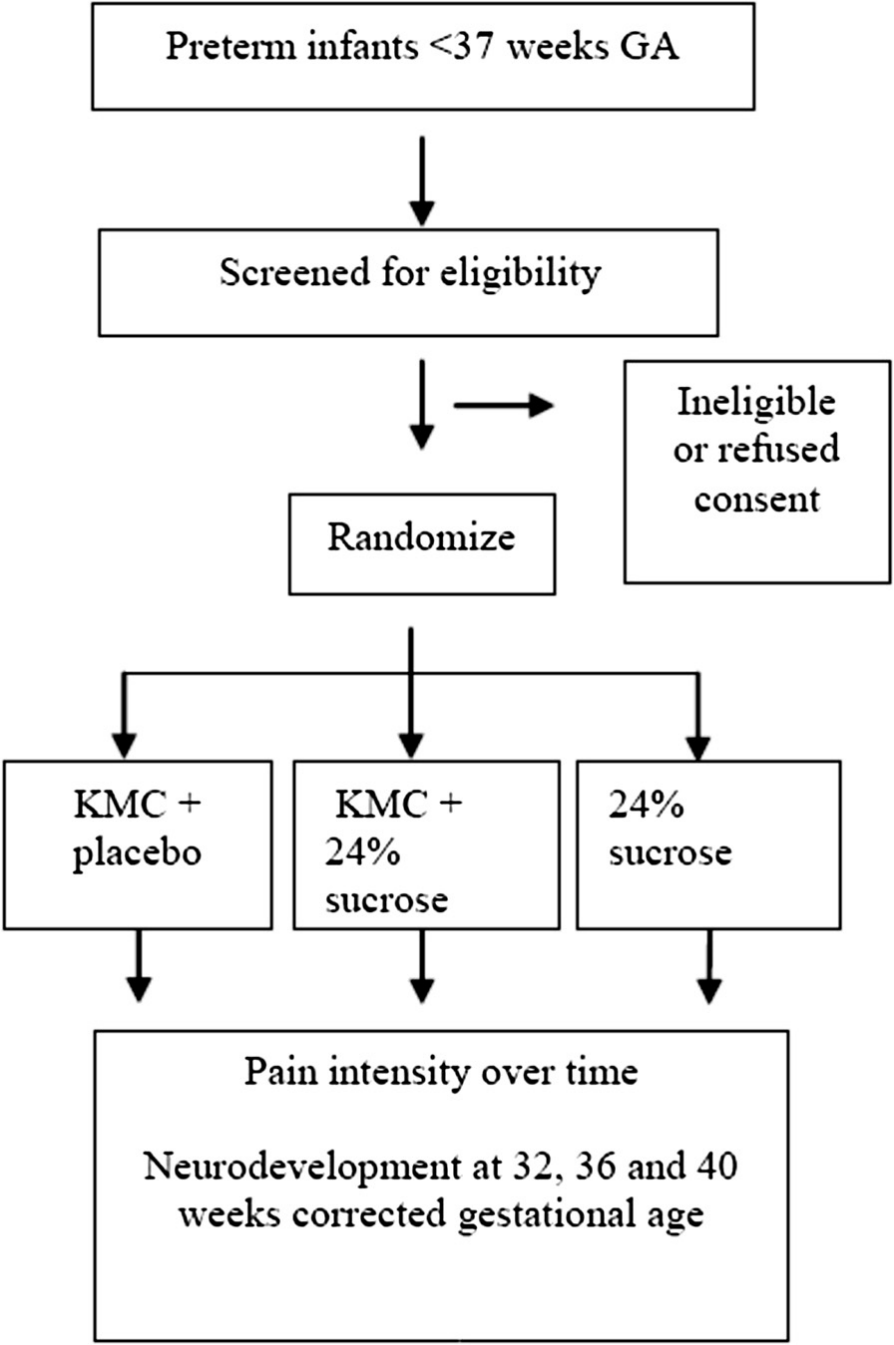
TRAKC Trial CONSORT Flow Diagram.

TRAKC is a single-blinded, randomized, parallel group design (Figure 1). The coders assessing the data will be blind to group assignment; however, the person completing the blood sampling will not be blind as it is impossible to prevent them from knowing participant KMC condition (i.e., skin-to-skin or incubator care).

### Participants

The sample will consist of 258 infants born less than 36 6/7 weeks gestational age according to the early ultrasound, that are stable enough to be held in KMC, whose mothers consent and expect to be available for KMC. Determination of infant physiological stability will be confirmed by the attending neonatal staff. Infant exclusion criteria are major congenital anomalies, receiving narcotics, or surgery. Infants will be enrolled within 5 days of birth.

### Sample size

The primary analyses of this study will be hierarchical linear modeling (growth curve analysis). Given that there are no acceptable methods for calculating sample size for this design, sample size estimates are based on Repeated Measures Analysis of Variance (RM-ANOVA). As a slope is being proposed in this study, with between group differences being specified, then across the slope (time), the between group differences will remain the same and thus the sample size should be the same at any point in time. In studies using RM-ANOVA, the sample size is based on group differences, and a similar principle applies. Given this, a sample of 258 will provide a power of 0.9 to detect a difference of 1.5 points (considered to be a clinically significant difference) with a standard deviation of 3 on the PIPP.

### Study setting

The study setting is the NICU of a tertiary-level Canadian hospital specializing in women and children’s health. Enrollment began in July of 2012, and is expected to be completed within 42 months of initiation.

### Randomization

#### Sequence generation

Group assignment will be determined using a password protected website, where randomization is in permuted blocks and participants will be stratified by gestational age (less than or greater than/equal to 32 weeks gestational age at birth) in order to ensure equal distribution across groups for all ages.

#### Implementation

Group assignment will be acquired by the research nurse from the secure website. The REB approved research nurse is the only individual in this study who has access to the secure site after consent is obtained.

### Allocation concealment mechanism

The pharmacy at the study site will supply the syringes containing the study solution. Of the study syringes, 1/3 will contain sterile water and 2/3 will contain 24% sucrose. The study solutions will be labeled with a code known only by pharmacy and the research nurse. Solutions will be prepared and packaged in an identical matter. Upon knowing infant group assignment, the research nurse will label each syringe with the infants study number and name to ensure added protection. Each participant will be provided syringes at his/her bedside and the research nurse will conduct regular syringe counts to evaluate compliance.

### Procedures

Documented approval has been obtained from the REB of the study site. When an eligible infant becomes available, the research nurse will explain the study to the parents, answer questions, determine the mother’s availability to provide KMC, and obtain written informed consent.

A minimum of 3 painful procedure sessions will be video-recorded with each infant, with each session being a minimum of 24-hours apart. For infants less than or equal to 32 weeks, sessions will be recorded as close to recruitment as possible, at 32 weeks, 36 weeks, and prior to discharge. For older infants, the sessions will be spaced as evenly apart as can be estimated. The research nurse will coordinate the timing of the procedure-recording with the staff, call parents to tell them that the procedure is taking place at a given time, and tell them the KMC condition (i.e., skin-to-skin or standard care).

The research nurse will mark phases on the recordings by holding colored cards briefly in front of the video camera. These phases will include baseline 1 at beginning of monitoring, baseline 2 prior to procedure initiation, heel warming or vein site cleansing, painful procedure (i.e., heel lance or venipuncture), application of bandage, and heart rate return to baseline. The research nurse will ensure that monitoring remains intact, and that the camera is focused on the infants face at an angle that makes the infant appear prone in an incubator in order to keep coders naïve [74]. Phases will be matched to the built in timer of the Somte monitor used to record physiologic data.

Depending on the gestational age of the infant, neurodevelopmental assessments using the Neurodevelopmental Assessment of the Preterm Infant will be conducted at 32 weeks, 36 weeks, and/or term equivalence by a trained research assistant who will be kept blind to group assignment.

Mothers will be asked to keep a diary about the amount of time they provide KMC regardless of group assignment. As one study has demonstrated that mothers stress in the NICU decreases with KMC [86], and as there is a plausible relationship between maternal cortisol levels and infant reactivity [87], there is an interest in studying maternal stress levels over time. Therefore, salivary cortisol samples will be collected from the mothers shortly after enrollment of the infant, halfway through the stay, and near NICU discharge. Samples will be collected near morning awakening in order to obtain basal cortisol levels, which are more reflective of trait anxiety as opposed to state anxiety. This data is exploratory, and will be correlated over time with infant pain response, accounting for group assignment. Mothers will also be asked 2–4 questions (depending on randomization) regarding their experience with providing KMC to their infants during hospitalization.

In addition to having mothers answer questions regarding KMC, nursing staff will also be questioned. At the beginning of the study, nurses will be asked to answer informal investigator questions aimed at assessing their attitudes toward KMC as an intervention for painful procedures. The questionnaires will be repeated every 6 months throughout the study. It is anticipated that nurses will have progressively more positive attitude toward KMC as an intervention as the study progresses. While attempts will not be made to sample the same nurses over time, the purpose of the questionnaire is to generate a sense of the units perceptions of KMC, and thereby understand what issues may arise in terms of fidelity to the study protocol.

### Intervention conditions

#### *KMC condition*

If the infant is randomized to the KMC condition, the research nurse will begin by recording a one-minute sample of the infant in the incubator. The research nurse will then place the mother and infant into the KMC condition, where the diaper-clad infant is help upright, at an angle of approximately 60°, between the mothers breasts, providing maximal skin-to-skin contact between mother and baby. The infant will remain in KMC for at least 15 minutes prior to the painful procedure. Two minutes prior to the procedure the infant will receive ¼ of the recommended (based on NICU protocol) volume of sterile water by syringe onto the tongue. The remainder of the total recommended dose will be given as needed in small increments during the procedure.

#### *Sucrose condition*

If the assigned condition is sucrose alone, half an hour before the procedure the nurse will place the infant in the supine position in an incubator or infant cot and set up the monitoring equipment. All monitoring will take place while the infant is in the incubator or cot. Two minutes prior to the procedure, the baby will receive ¼ of the recommended volume (based on NICU protocol) of 24% oral sucrose solution by syringe onto the tongue. The remainder of the total recommended dose may be given as needed in small increments during the procedure.

#### *Combined KMC and sucrose condition*

For combined sucrose and KMC, the infant will be placed in KMC as described for the KMC alone condition, however, will receive 24% oral sucrose solution as per the NICU protocol described above.

Although most painful procedures will not be video-recorded for the study analysis, the expectation is that the staff caring for infants enrolled in the study will follow the study intervention protocol for all painful procedures, and that any deviations from the study protocol will be charted and included in the analyses. Babies in the KMC group will have reminder signs at the bedside prompting the staff to place them in KMC for procedures, and the study syringes of either 24% sucrose solution or sterile water will be available to staff at the bedside throughout the infant’s stay in the NICU. Staff documentation of all painful procedures will be monitored to ensure compliance with the study protocol.

In order to ensure equipoise, staff may deviate from the protocol to give off-study doses of rescue sucrose if the infant has a PIPP score > 6 (considered to indicate pain) at their discretion. The deviations from protocol will be recorded on a procedure record kept at each infant’s bedside. If the mother is unavailable for a painful procedure, the infant will be swaddled and given the study solution. If the infant has a PIPP score > 6, off-study sucrose may be given by the nurse caring for the patient. All off-study sucrose doses will be recorded in the procedure record and the research nurse will confirm the doses given from the medication administration record.

### Outcomes

#### *Premature Infant Pain Profile (PIPP)*

The primary outcome in this trial will be infant pain intensity measured by the Premature Infant Pain Profile (PIPP) [72,88]. The PIPP uses seven indicators to calculate a composite pain measure: three behavioral indicators (facial actions of brow bulge, eye squeeze, and nasolabial furrow), 2 physiological indicators (heart rate and oxygen saturation), and 2 contextual indicators (gestational age and behavioral state) [72]. The contextual factors included in the PIPP are particularly important for this study as infants are being followed over time and therefore age changes will be accounted for. In addition, KMC is known to promote quiet sleep state, and this will be accounted for when considering behavioral state as an indicator in the PIPP scoring. The PIPP was developed 13 years ago and has steadily accumulated evidence of reliability and validity [72,88]. It had been used in over 40 studies to date [88].

This study will utilize a Somte (Compumedics, Melbourne, Australia) system that samples at a rate of 256 Hz to measure physiological data. Heart rate and oxygen saturation are recorded to the Somte system using a transcutaneous probe, and Electrocardiogram (ECG) is recorded from 3 leads. Epochs will be noted with an event timer in the Somte system. Close up video recordings of the infants face will be made using a Sony DCR-SR82 HDD digital video camera in the first baseline in the incubator (Baseline 1), one minute prior to start of procedure (Baseline 2), and during and after the procedure. The neurobehavioral state component of the PIPP score is determined according to Prechtl’s categories of quiet sleep, quiet awake, active sleep, or active awake [89], during the baseline [90]. Gestational age will be taken from the infant chart, and will be based on ultrasound at 16 weeks.

All data will be analyzed in the research laboratory outside the NICU. Physiologic data will be analyzed using Compumedics E-series Profusion PSG II software. Faces will be coded second-to-second on a stop frame system by trained coders naïve to the purpose of the study. Coders will be trained up to 90% agreement on video recordings from previous studies. Every three months, coders will recode randomly selected sessions (one from their earlier coding and one from each of the other coders) in order to determine both inter and intra-rater reliability.

#### *Neurobehavioral Assessment of the Preterm Infant (NAPI)*

The secondary outcome will be the Neurobehavioral Assessment of the Preterm Infant (NAPI), developed by Korner and colleagues [91-95]. In particular, the subscales of alertness and orientation and motor development and vigor will be assessed. The NAPI evaluates the relative maturity of functioning of preterm infants, with higher scores reflecting higher maturity, and can differentiate 2 weeks’ post-conceptual age. Assessments at 32 and 36 weeks corrected gestational age and at term equivalence will be preformed on all infants in accordance with their gestational age at birth, as well as at the time of discharge from the NICU. Much of the NAPI examination consists of observational items, and the remainder rates the infant’s response to stimuli. The assessment takes approximately 30 minutes to administer and includes clusters of single-item neurobehavioral dimensions. Test – re-test reliability ranging over 2 consecutive days ranged from 0.59 to 0.90. Original inter-observer reliability ranged from 0.64 to 0.93 [91]. Clinical and construct validity and sensitivity of the NAPI have been established [94,96].

### Other outcomes

Severity of medical risk at birth will be assessed by the SNAP-II (Simplified Newborn Illness Severity and Mortality Risk Score). Daily number tissue-damaging procedures will be recorded from the chart and the number of hours spent in KMC will be taken from a diary given to the mother. Mothers will also provide baseline salivary cortisol samples upon awakening on the days infants undergo a video-recorded medically required painful procedure.

### Statistical analysis

Analysis and inference will be based on the intention-to-treat principle. Descriptive and correlational analysis will be performed to identify potential confounding variables. The data will be analyzed using growth curve analysis. In this analysis, a regression line is fitted to each infant’s responses over time, and a slope and intercept is derived for each curve. The data will be centered so that the last time period is coded as 0, allowing for the comparison of the three groups, as well as the differences at final assessment. The advantage of this analysis is that 1) data can be missing as long as there are three data points, and 2) the spacing of the interventions does not have to be the same for all participants. This analysis will therefore answer all research questions: 1) what is the trend across time? (i.e., comparing slopes), 2) is KMC more efficacious than sucrose at reducing pain? (i.e., comparing the intercepts at the final assessment), and 3) is the combination of KMC and sucrose more powerful than either alone? Potential cofounders that may need accounting for in the statistical model include number of tissue damaging procedures, number of hours of KMC in total, number of doses of sucrose in total, SNAP-II score at birth, and gestational age at birth in days.

### Ethical considerations

Authorization and informed consent will be obtained from the mother of each eligible infant prior to study entry, and a copy of the consent form will be provided to participants once signed. Participation in the study is voluntary, and mothers will be made aware of their rights to withdraw their infants’ participation at any time throughout the course of the study. This study provides no direct benefit for the mothers or infants enrolled and compensation for study participation will not be offered.

Provision of KMC during painful procedures is not considered to be a standard of care in the NICU at the study site. While KMC is considered a safe practice for stable infants, all participants will be closely monitored with regard to any adverse events associated with being transferred into KMC. Administration of sucrose is considered standard care in the NICU at the study site. In order to ensure equipoise, NICU staff will be permitted to give off-study doses of sucrose if the infant has a PIPP score greater than 6, which is indicative of pain. Infants will be monitored as per NICU standard of care, and their clinical condition will be evaluated daily as part of medical rounds. All procedures that infants undergo will be part of their routine NICU care and will not be conducted solely for the purpose of this study.

## Discussion

This will be the first study to examine the repeated use of KMC for reducing pain response in preterm neonates. It is also the first to compare sucrose to KMC, or the interventions in combination, across time. Based on the theoretical framework of the brain opioid theory of attachment, it is fully expected that the results will be as hypothesized. However, current pain management guidelines are based on minimal data on repeated use of either intervention. Therefore, regardless of the outcomes of this study, results will have important implications for guidelines and practices related to management of procedural pain in preterm infants.

## Abbreviations

SSC: Skin-to-skin contact; KMC: Kangaroo Mother Care; NICU: Neonatal Intensive Care Unit; PIPP: Premature Infant Pain Profile; NAPI: Neurodevelopmental Assessment of the Preterm Infant; SNAP-II: Simplified Newborn Illness Severity and Mortality Risk Score.

## Competing interests

The authors declare that they have no competing interests.

## Authors’ contributions

MCY and CJ will take responsibility for overseeing all parts of the study. MCY will be responsible for the progress and timely completion of the trial. MCY, DI, and MV will oversee any issues related to KMC and neonatal care on the unit at which recruitment is taking place. ML will oversee the dissemination of research findings. CDW will oversee procedures related to collection and analysis of salivary cortisol samples. DS will assist with statistical design and analysis. BB and KC will oversee data collection and analysis. All authors contributed to manuscript development, and read and approved the final manuscript.

## Pre-publication history

The pre-publication history for this paper can be accessed here:

http://www.biomedcentral.com/1471-2431/13/182/prepub
